# A new double multiplication region method to design high sensitivity and wide spectrum SPADs in standard CMOS technologies

**DOI:** 10.1038/s41598-024-78070-6

**Published:** 2024-11-07

**Authors:** Utku Karaca, Ekin Kizilkan, Claudio Bruschini, Edoardo Charbon

**Affiliations:** Advanced Quantum Architecture Laboratory (AQUA), Ecole Polytechnique Fédérale de Lausanne (EPFL), 2002 Neuchâtel, Switzerland

**Keywords:** Imaging and sensing, Electrical and electronic engineering

## Abstract

Designing SPADs with high sensitivity in a wide wavelength range is crucial since the applications utilizing SPAD-based sensors target different parts of the spectrum. Here, we introduce a novel technique to achieve a wider sensitivity spectrum through the insertion of a second multiplication region into the depletion region. Thanks to the proposed method, at 5.5 V excess bias voltage, the fabricated devices achieved a PDP of 78% peak at 500 nm and 25.5% at 850 nm wavelength. At the same excess bias, we measured a normalized noise of 3.7 cps/μm^2^ and a jitter of 165 ps at 517 nm FWHM.

## Introduction

SPADs are p-n junctions operated above their avalanche breakdown voltage, in so-called Geiger mode; they are commonly used in optical detection owing to their low noise, high sensitivity, and high timing resolution^1,2^. Integration of SPADs with standard CMOS technologies has paved the way for the design of low-cost, large pixel arrays with embedded photon-counting and timestamping circuitry^1,2^. Applications, such as 2D imaging^3^, fluorescence lifetime imaging microscopy (FLIM)^4–6^, light detection and ranging (LiDAR)^3,7,8^, positron emission tomography (PET)^9,10^, and space imaging^11,12^ are currently driving the field. However, these applications demand high PDP at different wavelengths. For instance, LiDAR and space applications ask for near-infrared (NIR), whereas PET requires the detection of blue or ultraviolet (UV) photons. FLIM, on the other hand, can target visible to NIR spectra, depending on the emission wavelength of fluorophores. Therefore, separate SPAD designs are usually required, featuring high PDP at different parts of the wavelength spectrum and targeted uniquely at a specific application. However, a wide spectrum SPAD design would be able to address a number of applications, and its usage diversified. Thus, it is essential to design SPAD pixels with a wide spectral response so that they can be utilized in a variety of domains.

To achieve a wide spectrum SPAD, which has a high PDP from near-UV/blue to NIR, several issues have to be considered. Regarding the blue wavelengths, the depletion region of the SPAD should be close to the surface of the device since these photons are absorbed at very shallow thanks to the high silicon absorption coefficient of short wavelengths. To enhance the spectrum towards NIR, the depletion region could be extended deeper inside the epitaxial layer due to the reduced photon absorption at longer wavelengths^13–21^. Another alternative to collecting more photogenerated carriers at NIR is to ease the carrier diffusion by engineering the slope of the valence or conduction band depending on the carrier type through additional implants^19,22–24^. Although both techniques can provide well-performing SPADs, they have some drawbacks. In the wide depletion region approach, very high excess biases are required ($\sim$10 V) to be able to increase avalanche triggering probability and detection efficiency^25,26^. The magnitude of the electric field determines the electron-hole ionization coefficients and the probability of an avalanche. In a wide depletion SPAD, the electric field is lower at the breakdown voltage, and it rises slowly with increasing excess bias compared to a thin depletion device. Hence, the former requires very high excess bias to reach similar avalanche triggering probabilities as in the latter. High excess bias also requires voltage attenuation to adapt the voltage to a digital circuit’s low voltages. Moreover, wide depletion region SPADs might be noisier due to augmented Shockley-Read-Hall (SRH)-based thermal generation. Conversely, in the case of improving carrier diffusion, an additional n-type or p-type well is required to modulate the doping outside the depletion region of the SPAD and ease the diffusion of holes or electrons^21–24^. However, this can result in a longer diffusion tail, which might limit its use in timing-critical applications.

Obtaining a wide spectrum and low noise SPADs is challenging in more advanced CMOS technologies due to higher doping concentrations and reduced dimensions. As a result of higher doping values, shallower depletion regions are formed, which restrict detection wavelengths and deteriorate the overall noise performance due to augmented band-to-band (BTB) and trap-assisted tunneling (TAT) contributions. Therefore, device design advancements are required in order to realize high performance, wide spectrum SPADs for multispectral imaging based on advanced CMOS technology nodes as well.

In this paper, we present a new technique to improve the wide depletion region SPADs’ performances by creating two distinct multiplication regions in the same wide depletion zone. The technique, named *double multiplication region method*, achieves high PDP over an expanded spectrum and overall low noise, simultaneously for a large range of excess bias voltages. This enhancement in PDP at lower excess bias voltages is achieved thanks to the increased total avalanche triggering probability with the second multiplication region inserted. To the best of our knowledge, this is the first SPAD reported that has two multiplication regions in one depletion region. The proposed SPAD is different from the demonstrated dual-junctions SPADs^27^ in the sense that there is only one avalanche breakdown voltage and biasing configuration in the double multiplication region method. Dual-junction SPADs are basically a stack of two SPADs having two different breakdown voltages and thus biasing conditions. To prove the suitability of the technique in advanced CMOS technologies, we designed devices with an active area of 10 μm in a 110 nm CMOS Image Sensor (CIS) process using the standard existing doping implants. In fact, the proposed method can be applied to other CMOS technologies provided that a double-peaked p-well is present in the foundry process, which might not be the case at this point in time. The paper demonstrates the I-V curve, light emission tests, dark count rate (DCR), afterpulsing probability (APP), PDP, and jitter characterization results of the proposed device. The design methodology is also discussed and supported by numerical simulations in TCAD. The comparison of the device’s performance with several state-of-the-art wide depletion region SPADs found in the literature.

## Results

### I-V characterization and light emission tests

**Fig. 1:**
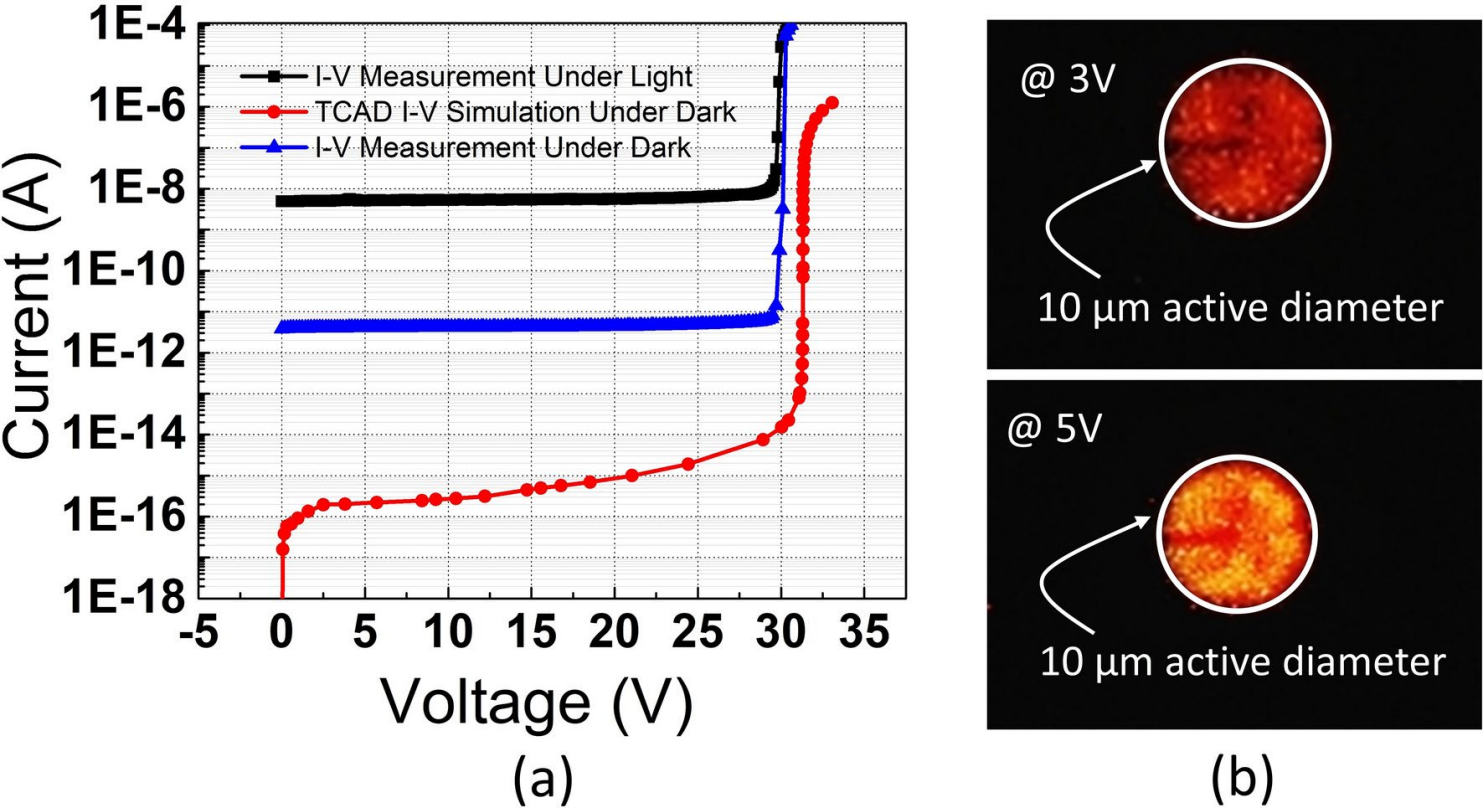
(**a**) Measured and simulated I-V characteristics of the proposed device. (**b**) Light emission tests at 3 V and 5 V excess bias voltages.

Fabricated devices were first characterized in terms of the I-V curve to demonstrate that avalanche breakdown exists in the proposed device. As shown in Fig. 1a, the avalanche breakdown occurs at 29.8 V under both dark and ambient light conditions. The relatively high breakdown voltage is an inherent consequence of designing a wide depletion diode, which requires a high bias to increase the electric field magnitude up to the critical breakdown field. Moreover, the breakdown voltage of the device in TCAD simulations in the dark was computed as 30.8 V. The small discrepancy between measurement and simulation may be due to some fabrication steps leading to differences in the real and the numerically implemented doping depth and concentration, or due to deviations of the electron and hole impact ionization coefficients in the fabricated silicon from the theoretical values utilized in the Okuto-Crowell model in TCAD. Then, Fig. 1b indicates the light emission tests of the device at two different excess bias voltages. The images prove that there is no edge breakdown in the proposed device, and the photoresponse is quite uniform over the active area.

**Fig. 2:**
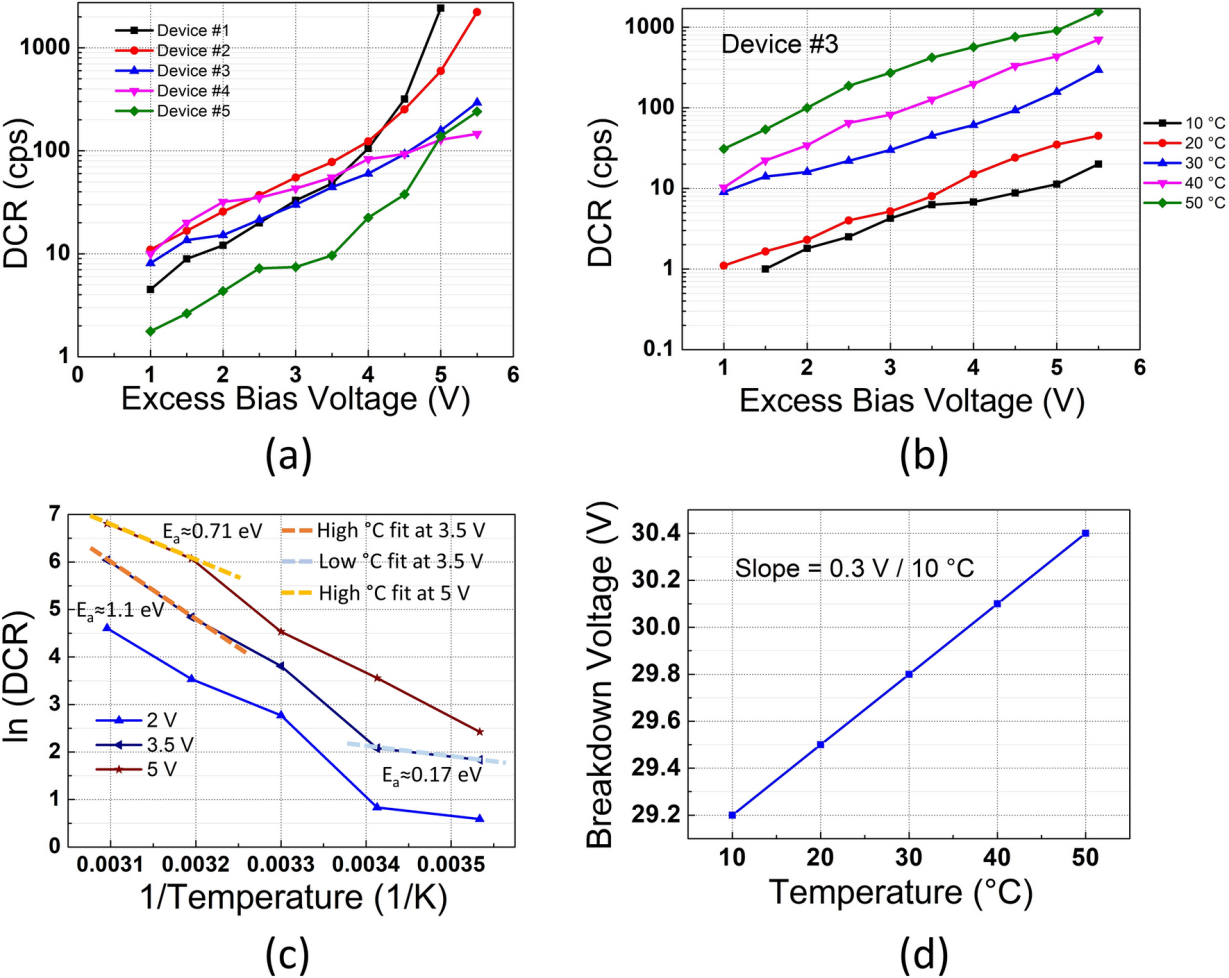
(**a**) DCR measurements of the device on 5 different dies for various excess bias voltages at room temperature. (**b**) DCR vs. temperature characterization of the selected Device #3. (**c**) Arrhenius plot of the selected device with the indicated trap activation energy at high and low temperatures. (**d**) The change in breakdown voltage between 10 and 50 °C.

### Dark count rate

The noise of the device was measured on five separate dies to understand its variation on the same wafer. As the devices were manufactured in a multi-project wafer (MPW) run, the number of devices is limited. DCR results at room temperature from these five dies are provided in Fig. 2 (a) with respect to the applied excess bias voltage. It can be noted that DCR is quite uniform up to 4 V excess bias, whereas DCR might increase drastically after 4 V. In all the devices, a significant increase in DCR was observed after 5.5 V excess bias, which might be due to high tunneling or afterpulsing. Device #3 was then selected to illustrate further characterization since it corresponds to the median of these DCR measurements.

Figure 2 (b) shows the DCR of Device #3 as a function of temperature. Between 10 and 20 °C noise is dominated by tunneling generation, which is affected primarily by excess bias. Starting from 20 °C, thermal generation begins to dominate, while at 30 °C, the device reaches 61 cps at 4 V and 295 cps at 5.5 V excess bias voltages. The corresponding normalized DCRs by the SPAD active area are 0.76 cps/μm^2^ and 3.7 cps/μm^2^ at the same excess biases. Due to the fact that the proposed device has two multiplication regions and enhanced avalanche breakdown probability, the DCR might be higher than the reported wide spectrum SPADs at the same excess bias voltage. Above 30 °C, DCR increases significantly with thermal generation, attaining 570 cps and 1560 cps at 4 V and 5.5 V excess bias voltages, respectively, at 50 °C. Furthermore, in order to verify the contribution of thermal and tunneling generation to the DCR, an Arrhenius plot is depicted in Fig. 2c. It is found that at temperatures above 20 °C, the trap energy level is around 1.1 eV at 3.5 V excess bias voltage. This activation energy corresponds to the bandgap of the silicon, which means that thermal generation is dominating the noise. Below 20 °C, an activation energy of 0.17 eV was obtained, meaning that trap-assisted tunneling is dominating DCR due to the reduced thermal generation at lower temperatures. Furthermore, at 5 V excess bias voltage, 0.71 eV activation energy was observed above 20 °C. This is because the tunneling noise is affected and enhanced by the voltage, but it stays rather insensitive to the temperature; thus, the tunneling noise becomes comparable with the thermal generation at high excess bias voltages, and an activation energy less than the bandgap was achieved.

Breakdown voltage change with temperature is illustrated in Fig. 2d. It is found that at each 10 °C, the breakdown voltage varies by about 300 mV, and the curve follows a virtually ideal behavior such that the breakdown voltage decreases while decreasing the temperature due to the reduced phonon scattering.

### Afterpulsing probability

**Fig. 3:**
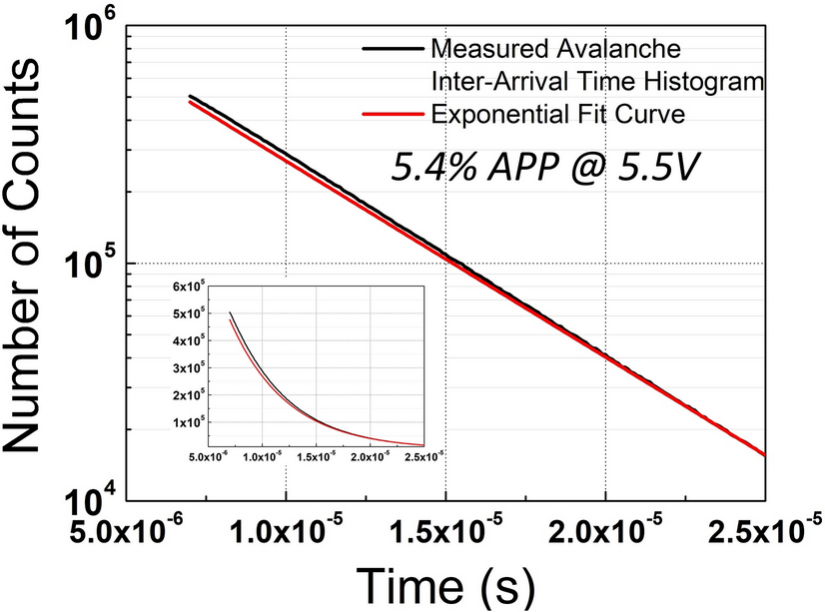
Inter-arrival time histogram of the device at 5.5 V excess bias voltage.

Afterpulsing is crucial in many time-sensitive experiments since correlated noise can adversely affect the DCR and PDP of SPADs. In principle, when an avalanche occurs, large numbers of carriers are generated in the depletion region, while they might be trapped in defects of the material. The release of trapped carriers after relaxation creates additional unwanted pulses, which are called afterpulses. To calculate the APP of the device, a histogram of the measured inter-arrival times of the SPAD pulses was measured, as in Fig. 3. Because of passive quenching and recharge, the dead time of the device was identified to be relatively long in our device, around 7 μs. The dead time was determined when the count difference between the measured and fit curve reached a plateau and started to drop after that point. A fitting procedure was performed by considering the fact that inter-arrival time behavior follows the Poisson statistics in the absence of afterpulsing, which is depicted by the red curve in Fig. 3. For the fitting, the time interval between 15 and 25 μs was chosen, where the histogram follows an ideal exponential function. Any deviation from the fit curve basically represents afterpulsing. The APP is defined as the area between the measured and fitted curves divided by the total area under the fit. The APP was calculated to be 5.4% at 5.5 V excess bias voltage.

**Fig. 4:**
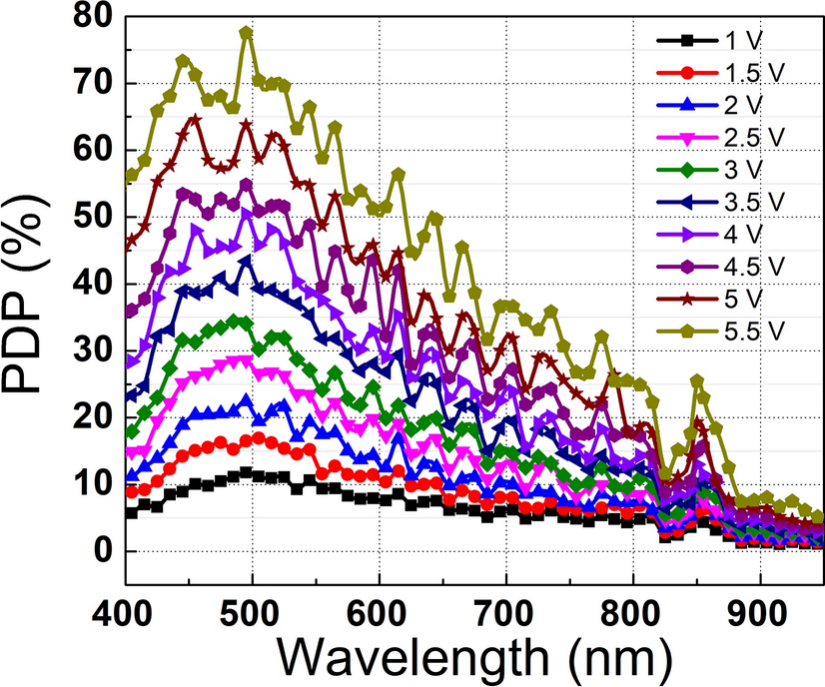
PDP measurements of the device for various excess bias voltages.

### Photon detection probability

The measured PDP curves from 1 V to 5.5 V excess bias voltage and from 400 nm to 950 nm wavelengths are shown in Fig. 4. In the calculation of PDP, the area observed in the light emission tests was used, which has a 10 μm diameter as shown in Fig. 1 (b) and corresponds to the active area of the device. As illustrated in the figure, the maximum PDP achieved at 5.5 V excess voltage is 78% at 500 nm. Since the first multiplication region is located very close to the surface, it favors the avalanche triggering of electrons generated from short-wavelength photons. The photogenerated holes at short wavelengths can also go through the avalanche multiplication owing to the second multiplication region inserted to the far edge of the depletion region. Furthermore, the PDP is also enhanced at NIR wavelengths, such that it reaches 25.5% at 5.5 V excess bias and 850 nm, thanks to the wide depletion region formed and the enhanced avalanche breakdown probability for photogenerated electrons that are diffusing to the depletion region. Since the device is substrate-non-isolated, many carriers can diffuse to the junction, where the diffused electrons have more chances to trigger an avalanche with a double multiplication structure. Another property of the structure improving the NIR PDPs was to have a p-substrate with a graded doping profile, which also enhanced the diffusion of the photogenerated electrons from the substrate to the depletion region. The doping profile of the substrate cannot yet be disclosed due to the confidentiality of the foundry process. In addition, some sudden PDP drops in the NIR were also observed, which might be due to the not yet fully optimized dielectric stack on the SPAD, which causes optical reflections.

**Fig. 5:**
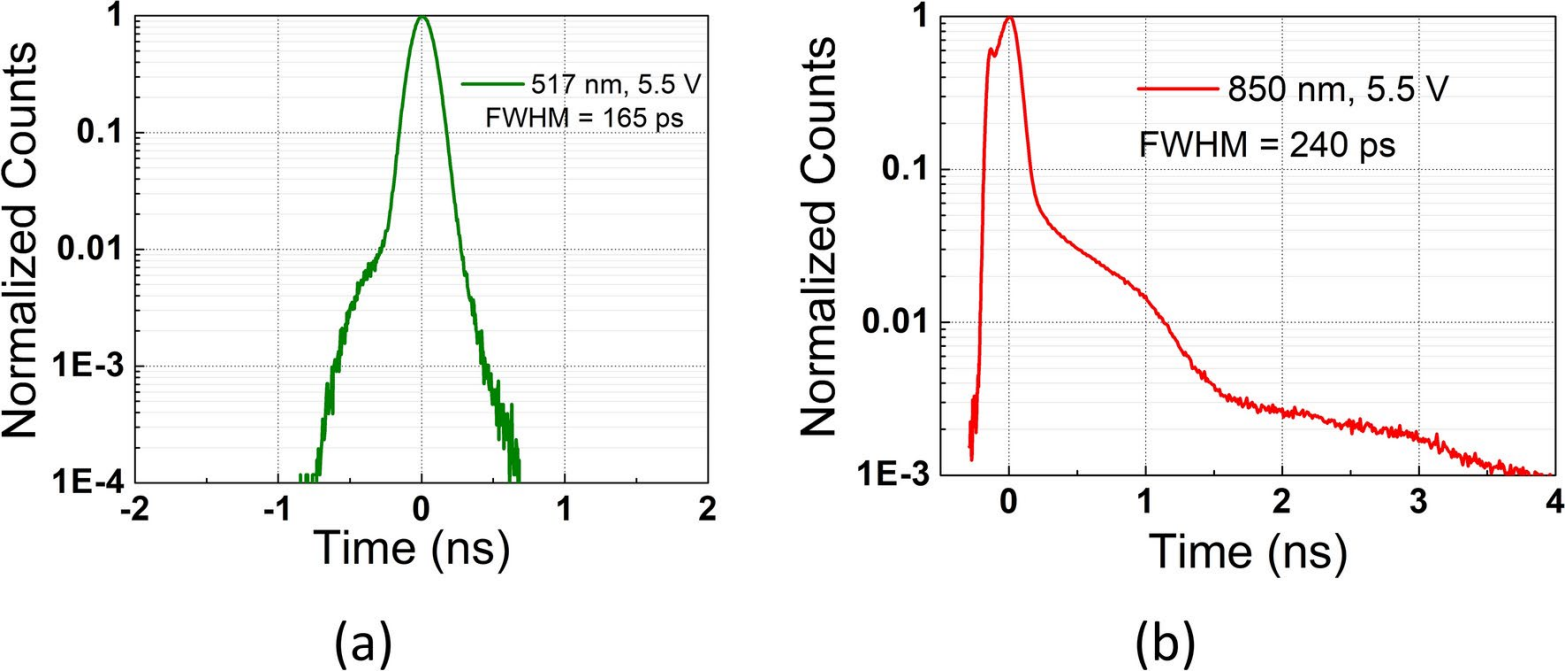
Jitter measurements of the device (**a**) at 517 nm and (**b**) at 850 nm and at 5.5 V excess bias voltage.

### Timing jitter

In this work, the timing histograms of the device were obtained with pulsed lasers operating at 517 nm and 850 nm wavelengths. Figures 5a,b show the histograms belonging to these two wavelengths, while jitter is indicated as full-width-at-half-maximum (FWHM). At 517 nm, the jitter is obtained as 165 ps, whereas at 850 nm it is calculated as 240 ps, both at 5.5 V excess bias voltage. Jitter measurements were taken in the single-photon regime ($\approx$ 0.1 photon per pulse), while the laser was operating at 100 kHz repetition frequency. Due to the many detected diffused electrons with the substrate-non-isolated SPAD structure, a diffusion peak was observed in the timing histogram at 850 nm^28^. The first peak, with a smaller magnitude and width, corresponds to the contribution of the depletion region, whereas the second peak and very long diffusion tails represent the detected diffused carries from the substrate. Since the number of detected photons through the diffusion process is greater than the ones absorbed in the depletion region, a deteriorated timing jitter of 240 ps FWHM was measured at 5.5 V_ex_ in the device. These results indicate that the proposed design is suitable for use in highly timing-critical applications.

## Discussion

**Fig. 6:**
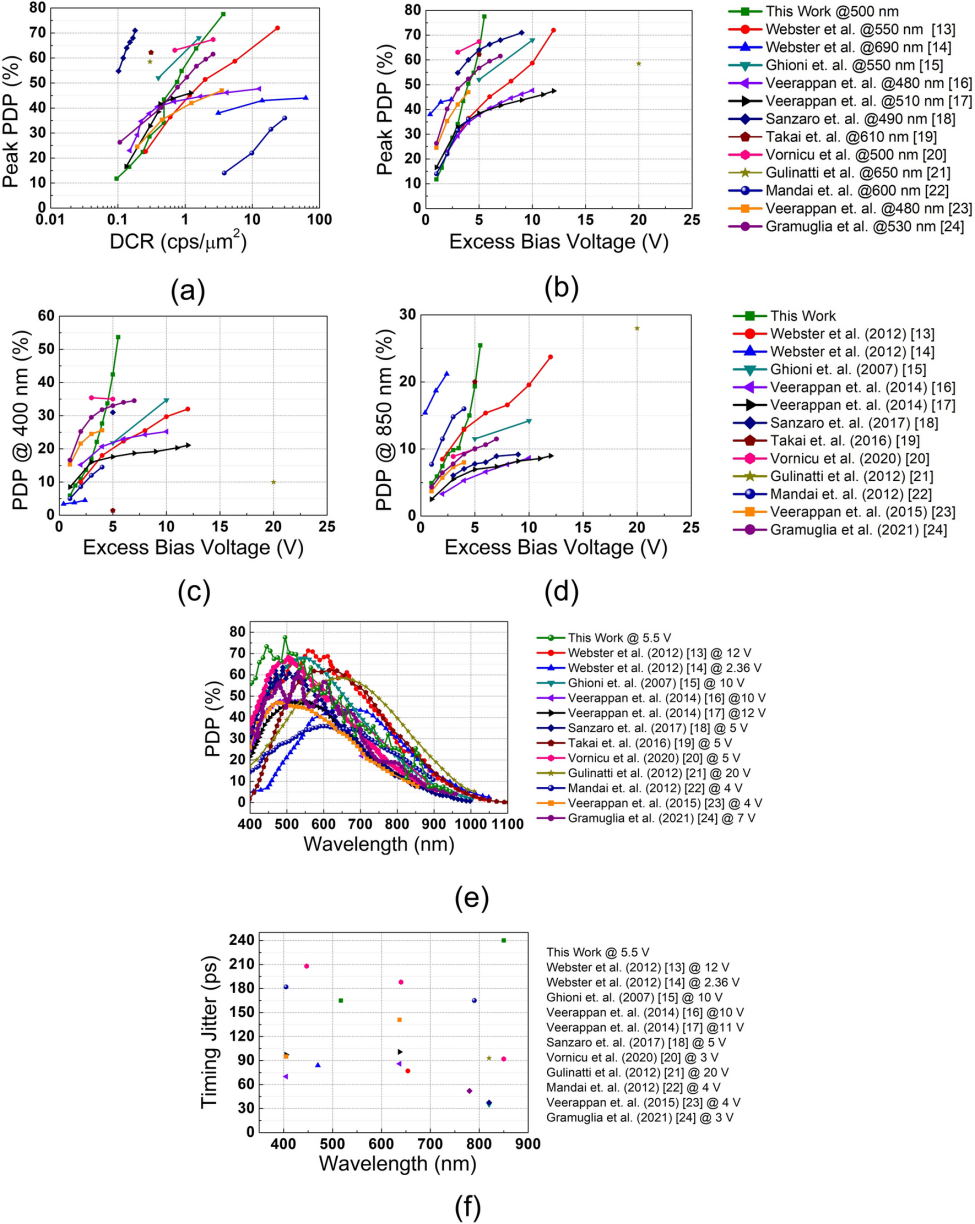
Comparison of the proposed device with state-of-the-art FSI wide depletion CMOS SPADs according to (**a**) peak PDP versus normalized DCR with the active device area, (**b**) peak PDP at the measured excess bias voltage, (**c**) PDP at 400 nm versus excess bias voltage, (**d**) PDP at 850 nm versus excess bias voltage, (**e**) PDP spectrum at the highest excess bias voltage, and (**f**) timing jitter at measured wavelengths and at the highest excess bias voltage.

### Comparison with the state-of-the-art

To demonstrate the effectiveness of the double multiplication region method, we compared DCR and PDP with previous works in Fig. 6. For this purpose, we chose front-side-illuminated (FSI) wide depletion region CMOS SPADs found in the literature. In Fig. 6a, peak PDP is plotted with respect to the normalized DCR in accordance with the active area of each device. It shows that the noise of our device mostly follows the same trend as the other devices, even though there are also a few extremely low-noise devices, such as^18^. However, it can be observed that our device reaches the highest PDP, still with one of the lowest noise levels. Even if the second multiplication region can increase the DCR of the device, the noise of our device is comparable to or better than the wide spectrum SPADs at the same PDP level, except^18,19,21^. Moreover, in order to prove that we have eliminated the need for high excess bias in the wide depletion region devices, peak PDP is depicted at the measured excess bias voltage in Fig. 6b. As can be seen in this graph, the common trend is to bias the SPADs around 10 V or above to obtain a high PDP. On the other hand, we achieved a high PDP only at around 5.5 V excess bias without deteriorating DCR thanks to the second multiplication region, which increases significantly the probability of having avalanche multiplication.

Since the main purpose of this study is to realize a wide spectrum SPAD that can be effectively operated from blue to NIR for various applications, the PDP of the proposed device is also compared with the state-of-the-art at 400 nm and 850 nm wavelengths, as shown in Fig. 6c,d, respectively. At 400 nm, our device attains 53.5% at 5.5 V excess bias, which is the highest among the FSI wide depletion region SPADs. At 850 nm, we also reach one of the highest PDPs of 25.5% at 5.5 V excess bias, where the maximum value achieved is 28% in^21^. Ultimately, the PDP spectrums of the proposed SPAD and state-of-the-art wide spectrum SPADs are compared in Fig. 6e. Since the first multiplication region is very close to the surface and both carriers can be multiplied, the proposed SPAD achieved better sensitivity below 550 nm. For longer wavelengths, there are better-performing devices thanks to designs with deeper junctions and wider depletion regions. Nevertheless, the performance of our new device is comparable with the state-of-the-art between 750 nm and 850 nm thanks to the wide depletion region and double multiplication structure, resulting in improved total avalanche triggering probability. Finally, the timing jitter comparison with the state-of-the-art is provided in Fig. 6f. The jitter of the new SPAD is at an acceptable level for timing-critical applications.

### Conclusion

In this study, we developed a new technique that we call *double multiplication region method*, to design low-noise and wide spectrum SPADs, suitable for applications requiring time-resolved photon-counting sensitivity at a broad wavelength spectrum. To the best of our knowledge, this is the first SPAD device that has two multiplication regions in the same depletion, thus having only one breakdown voltage and biasing condition. Owing to our new method, we eliminated the need for very high excess bias in wide depletion region SPADs, which could make integration with supporting circuits more complicated. Although the devices were fabricated and simulated in a 110 nm CIS technology, the technique can be applicable to any CMOS technology, provided that an existing layer has a double-peaked doping concentration and can create an n-p-n-p type doping profile in the SPAD structure. We also conducted extensive TCAD modeling that showed that two distinct multiplication regions are formed in a merged, wide depletion region of the proposed device. Moreover, the avalanche breakdown probability simulations reveal that the electrons and holes can go through the avalanche multiplication simultaneously at two different multiplication regions for the carriers generated in the depletion region. Regarding the carriers diffusing to the depletion, they have more chance to trigger an avalanche, thanks to the second multiplication region. Therefore, an improved avalanche breakdown probability, and as a result, an enhancement in the PDP of the SPAD were obtained. The fabricated devices achieved 78% peak PDP at 500 nm wavelength and 5.5 V excess bias voltage. For this PDP level, we obtained a normalized noise of 3.7 cps/μm^2^ and 5.4% afterpulsing probability. At blue wavelengths, the device reached 53.5% PDP at 400 nm. At NIR, the SPAD achieved 25.5% PDP at 850 nm at the same excess bias voltage. These are among the highest PDP and lowest noise values reported in wide spectrum CMOS SPADs. The jitter of the SPAD was measured as 165 ps FWHM at 517 nm and 240 ps FWHM at 850 nm. The results indicate that the double multiplication region method is an effective way of designing CMOS SPADs suitable for a number of time-resolved photon-counting applications requiring wide spectra of operation.

## Methods

### Device design in TCAD

In our study, to address the high excess bias need of wide depletion region devices, we analyzed the electric field simulations that were provided in the previous works^13–24^. As illustrated in these articles, in the depletion region, there is only one multiplication region for carriers, where the high electric field exceeds the critical breakdown field (3x10^5^ V/cm for silicon). The existence of one multiplication region enables the impact ionization of either photogenerated electrons or holes due to electric field direction, which limits the PDP of the devices. Following this analysis, we developed a series of standard implants to achieve a double multiplication region. Double multiplication provides two advantages: (a) for the photons absorbed inside the depletion region, both carriers will have the chance to trigger an avalanche; (b) for the photons absorbed outside the depletion region, the electrons reaching the depletion will have the chance to undergo the avalanche process two times. Therefore, independent of the absorption location, the total avalanche breakdown probability can be increased for a photogenerated electron-hole pair, leading to a significantly higher PDP.

**Fig. 7:**
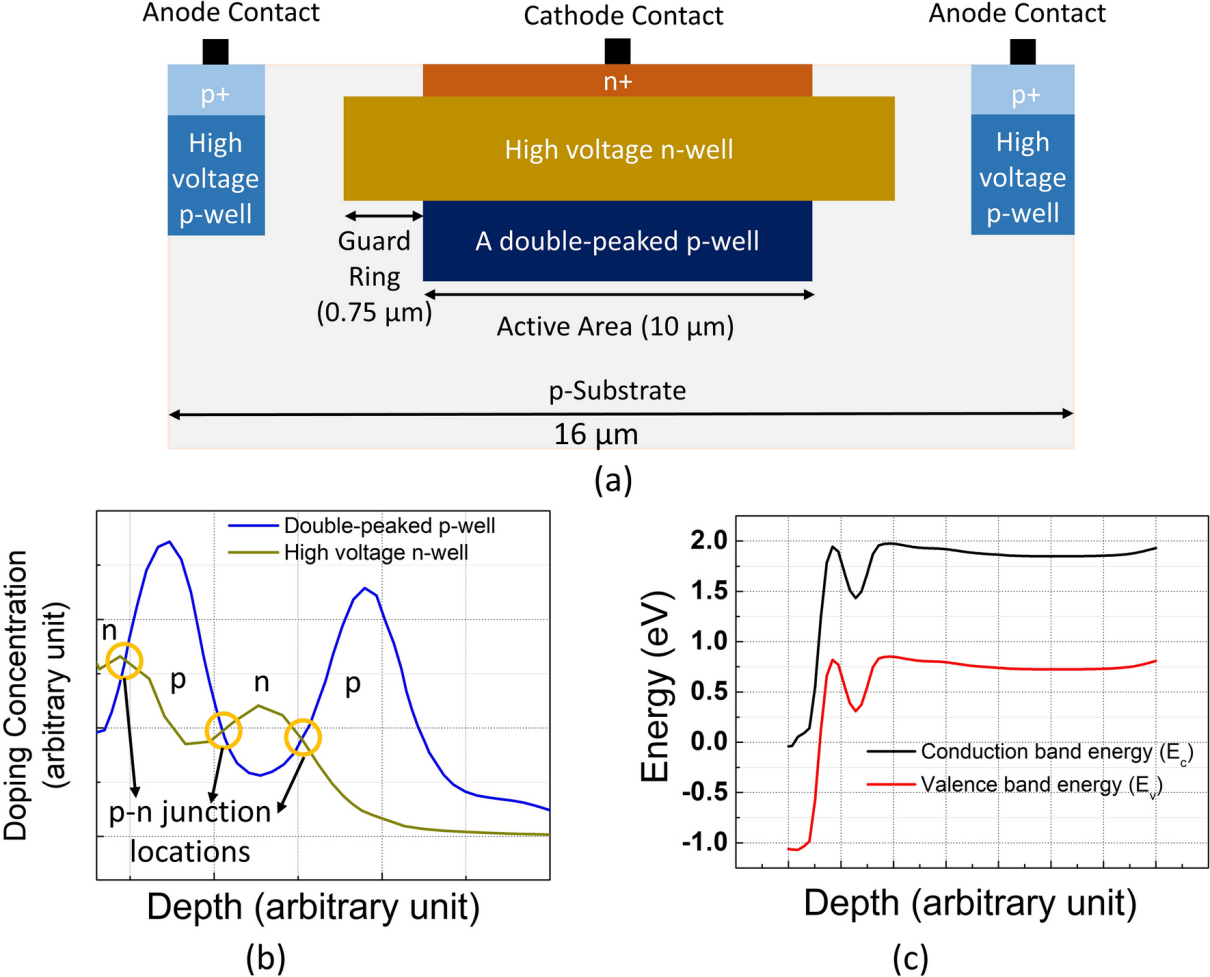
(**a**) Cross-section of the proposed device, (**b**) Doping profiles of the utilized layers in arbitrary units, and (**c**) The energy band diagram of the structure at 1 V reverse bias voltage.

The cross-section of the proposed device can be seen in Fig. 7a. As indicated, a high-voltage n-well and a double-peaked p-well layer were chosen to design the SPAD. The active area defined by the p-well has a 10 μm diameter, and there is a 0.75 μm virtual guard ring to prevent edge breakdown. From contact to contact, the whole SPAD covers 16 μm diameter. Besides, the doping profiles of the utilized layers are depicted in Fig. 7b. The superimposed doping concentrations show that there are in fact three p-n junctions, which are marked with the yellow circles on Fig. 7b. The first junction is an n-p, the second one is a p-n, and the third one is an n-p junction, as can be seen from the device surface perspective. The characterization of the device was performed in reverse bias mode, such that a high voltage was applied to the n^+^ side of the first junction and the p-side of the third junction was kept at zero volts via grounding the p^+^. In this biasing scheme, the first junction becomes reverse biased, the second is forward biased, and the last one also becomes reverse biased. Since the second p-n type junction is actually forward biased, it does not contribute to the avalanche and does not have an avalanche multiplication region. Therefore, it is expected that there are two multiplication regions in our device, which are separated by a forward-biased p-n junction. Furthermore, at such high voltages to reach avalanche breakdown, it was thought that all depletion regions could be merged to form one wide depletion region, where the electric field magnitude would never reach zero since the forward-biased junction would stay very thin. To demonstrate the existence of three different junctions, an energy band diagram of the device at 1 V reverse bias voltage is also given in Fig. 7c. As shown, there are two positive-sloped regions corresponding to the n-p junctions and one negative-sloped region belonging to the forward-biased p-n junction. This simulation further verifies that only two multiplication regions occur in our device, and they might fit into the same depletion region if the depletion region merge happens at the breakdown voltage. Figure 7 overall indicates that having a second peak in the p-well is necessary to achieve an n-p-n-p type junction and to form a second multiplication region so that the technique can be used in other CMOS technology nodes as well.

To further support our claim that two distinct multiplication regions exist in our device, we conducted numerical simulations in the TCAD environment^29^ by integrating the precise doping profiles of implant layers provided by the foundry. In TCAD, coupled Poisson and electron-hole drift-diffusion equations were solved with the SRH recombination mechanism. Carrier recombination rates are calculated by defining electron and hole recombination lifetimes. The TAT contribution in I-V was computed via the Schenk model^30^, which modifies the lifetimes under high electric fields. The Okuto-Crowell model was used to identify the avalanche breakdown voltage in silicon^31^ and the McIntyre model was utilized to determine position-dependent avalanche triggering probabilities^32^.

**Fig. 8:**
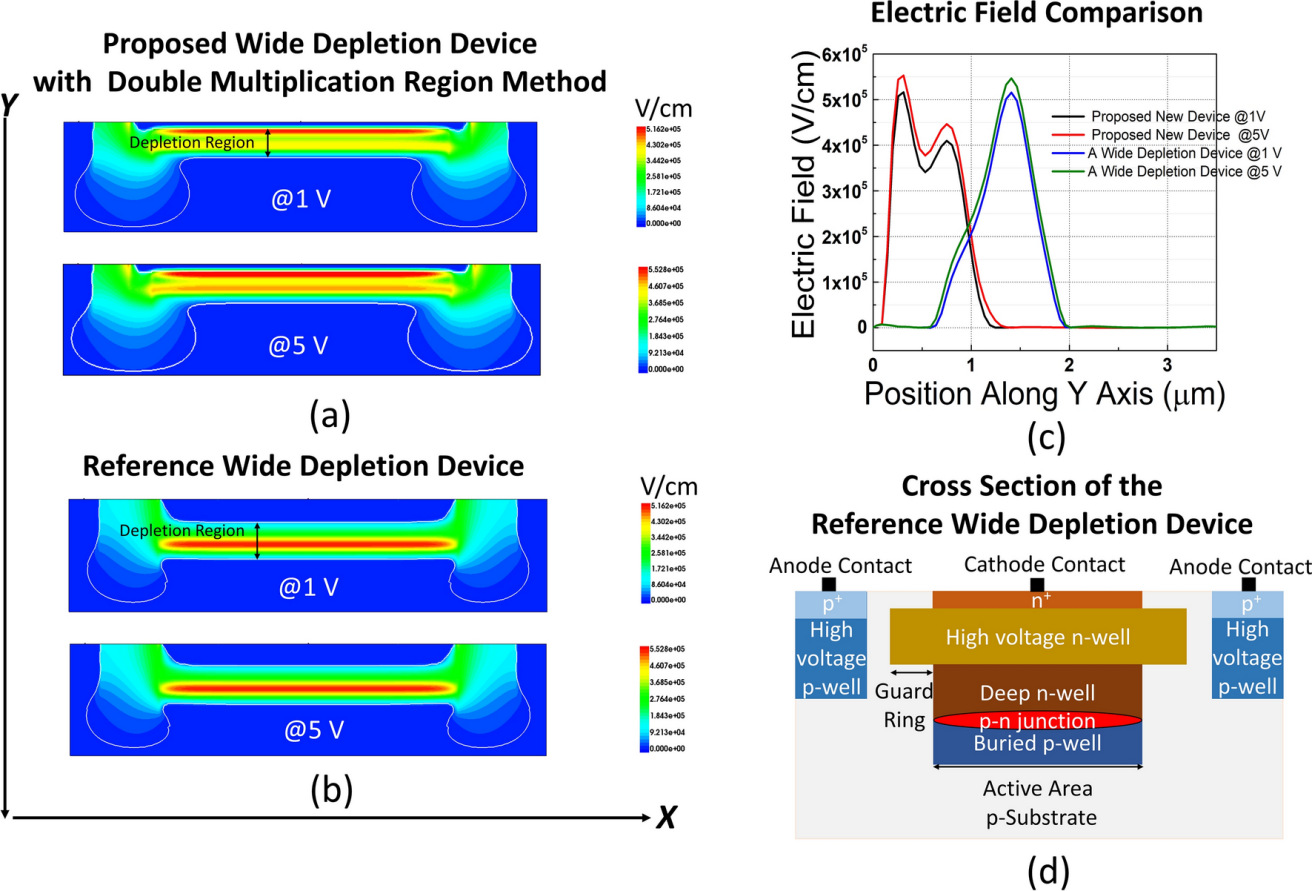
Electric field profiles of (**a**) the proposed device with the double multiplication region method and (**b**) SPAD with a wide depletion. *Note*: White lines correspond to the depletion region boundaries. (**c**) The magnitude of the electric field at the center of each device along the y axis. (**d**) The cross-section of the reference wide depletion SPAD.

Under these conditions, as a reference, we first replicated the previously designed wide depletion SPADs in the literature by simulating a SPAD using the technology’s available layers. The cross-section of the wide depletion SPAD example is provided in Fig. 8d. The structure is based on high-voltage n-well/deep n-well/buried p-well layer implants, with the junction located between the deep n-well and the buried p-well. It is similar to the SPAD design demonstrated in^13^, except that the buried p-well is used in the junction instead of the p-epi layer. This structure with n^+^ contact on top of the SPAD area is preferred to be able to sweep the carriers in the same direction as in the proposed SPAD. It will thus not have an effect on the avalanche breakdown probability simulations due to the impact ionization coefficient differences between the electrons and the holes. The breakdown voltage of this reference SPAD is 31.5 V as simulated in TCAD. The electric field simulation results of the reference wide depletion SPAD are seen in Fig. 8b, where the area highlighted in red corresponds to the multiplication region. As shown in the example device at both 1 V and 5 V excess bias, the depletion region contains only one multiplication region, and in the remaining space, the electric field decays towards the edges. Since this device suffered from edge breakdown after fabrication, the characterization results of this device could not be included.

Then, the proposed design was simulated, which has a 30.8 V breakdown voltage and a slightly (0.1 μm) shorter depletion region than the reference wide depletion SPAD. The electric field results of the proposed SPAD are provided in Fig. 8a. As can be seen, there is only one depletion region, whose boundaries are set by the white lines and highlighted with a black arrow. As expected, we have a merged, single wide depletion region at such high reverse bias voltages. This merging occurs because the depletion region width of the forward-biased p-n junction between the multiplication regions is quite narrow. Around x = 0.4 μm, the electric field decay occurs in the forward-biased region, where a lot of injected carriers cause a charge drop. However, the field rises again before reaching zero, as can be observed in Fig. 8c, which is taken at the center of the devices along the y axis. This rise in the electric field, with the second peak in the p-well, forms the second multiplication region. Hence, the existence of two multiplication regions is also proven by the two peaks appearing in the electric field simulations. At 1 V excess bias, in addition to the first multiplication region shown in red, the second multiplication region is highlighted in yellow, where the electric field is also above the critical field for avalanche breakdown to occur. At 5 V excess bias, the second multiplication is painted in orange, and it shows that the electric field stays above breakdown in almost the entire depletion region, in contrast to reported wide depletion SPADs^13–24^.

By interpreting Figs. 7b and 8a further, it can be verified that the first multiplication region occurs at the first intersection point of the p- and n-wells. Then, the electric field magnitude decreases around the second p-n junction location. Finally, the second multiplication region is located starting from the third intersection point between these two wells. The second multiplication region is prominent in our proposed device, with the emerging second peak in Fig. 8c. Thus, by keeping the first multiplication region close to the surface, we aim to increase the PDP at short wavelengths. Furthermore, by constituting a wide depletion region and inserting the second multiplication close to the deep edge, we aim to enhance the PDP at longer wavelengths by giving the electron two chances at which they can undergo the avalanche process. Regarding the moderate wavelengths, photogenerated electrons can get multiplied at the upper, and holes can get multiplied at the lower multiplication regions. Ultimately, by having multiplication centers at both edges of the depletion region, we expect that the electron and hole avalanche triggering probabilities will be maximized. As a consequence, the DCR of the SPAD might also increase since thermally or tunneling-assisted generated carriers will have a higher probability of triggering an avalanche pulse.

**Fig. 9:**
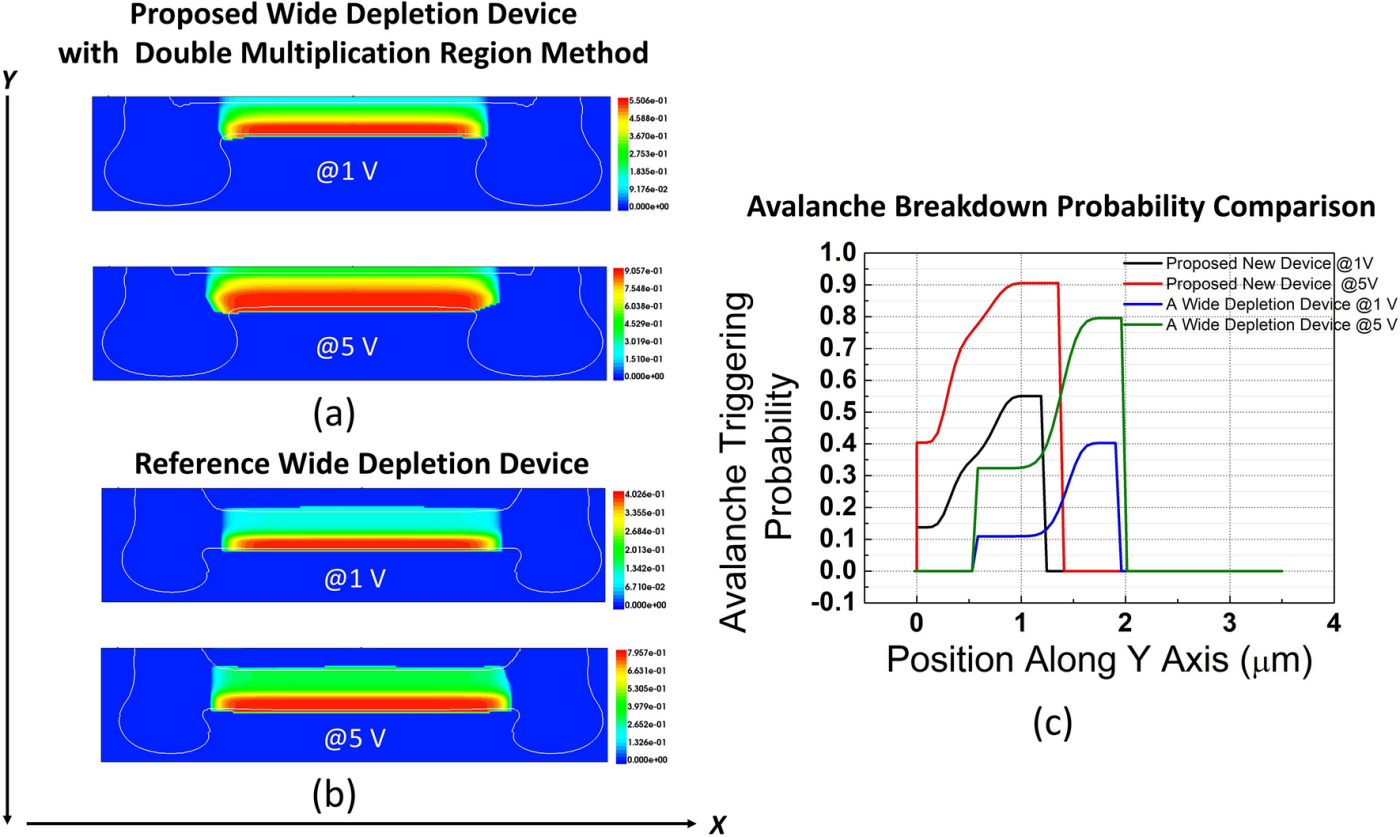
Electron-hole avalanche breakdown probabilities of (**a**) the proposed device with double multiplication region method, and (**b**) reference wide depletion SPAD. (**c**) The values of the avalanche breakdown probabilities at the center of each device along the y axis.

Along with the electric field profiles, we also computed the avalanche breakdown probabilities of our new device and the same reference wide depletion SPAD. In order to solve the breakdown probabilities, as mentioned before, McIntyre model^32^ was implemented in TCAD, which has the following formulations:

1 $$\begin{aligned} \frac{dP_{\text{e}}}{dx} = (1-P_{\text{e}}) \; \alpha _{\text{e}} \; P_{\text{j}},\end{aligned}$$

2 $$\begin{aligned} \frac{dP_{\text{h}}}{dx} = -(1-P_{\text{h}}) \; \alpha _{\text{h}} \; P_{\text{j}} \end{aligned}$$

3 $$\begin{aligned} P_{\text{j}} = P_{\text{e}} + P_{\text{h}} - P_{\text{e}}P_{\text{h}} \end{aligned}$$

under the boundary conditions $\hbox {P}_{\text{e}}$(0)=0 and $\hbox {P}_{\text{h}}$(Depletion width)=0, where $\hbox {P}_{\text{e}}$, $\hbox {P}_{\text{h}}$ and $P_{\text{j}}$ are the probabilities of avalanche breakdown for electron, hole, and electron-hole pair generated at position x. Additionally, $\alpha _{\text{e}}$ and $\alpha _{\text{h}}$ represent the impact ionization coefficients for electron and hole, which are modified by the magnitude of the electric field according to the Okuto-Crowell model^31^. Equation 1 and Equation 2 demonstrate the variation of the electron and hole avalanche breakdown probabilities with respect to the position, and Equation 3 indicates the joint breakdown probability for an electron-hole pair. Based on these models, the joint breakdown probability of each device was calculated in TCAD, and the results are provided in Fig. 9. The second multiplication region in the proposed device, as illustrated in Fig. 9a, significantly increases the volume of avalanche breakdown probability from 1 V to 5 V excess bias, reaching 90% in half of the depletion region. In contrast, in the reference wide depletion device, as shown in Figure 9 (b), the high avalanche breakdown probability is restricted to the only existing multiplication region, and it rapidly decreases and remains low in a significant portion of the depletion region. The precise values of breakdown probabilities at the centers of the devices along the y axis are given in Fig. 9c.

To explain the findings more clearly, let us imagine the absorption of a photon in the middle of the depletion region in both devices. In the reference wide depletion SPAD at x = 1.2 μm, only the photogenerated holes will go through the impact ionization process in the multiplication region centered at x = 1.4 μm. However, the electrons will not get multiplied due to low electric fields. Conversely, in the proposed double multiplication region SPAD at x=0.6 μm, both the holes and the electrons will be multiplied in the multiplication regions centered at x = 0.75 μm and at x = 0.3 μm, respectively. Due to the lack of a multiplication region for the electrons, the total avalanche breakdown probability obtained for the reference wide depletion SPAD is only 33% in the middle of the device, due to the contribution of the only holes, whereas it reaches 77% in the proposed SPAD, as can be observed from Fig. 9c, thanks to the existence of the second multiplication region. Moreover, regarding mostly the longer wavelengths, the diffusing electrons from the substrate have 80% chance of getting multiplied in the reference wide, whereas it is enhanced to 90% in the proposed device, as calculated from values at the lower edge of each depletion region. This means that the photo-generated electron-hole pairs produced in the depletion or reaching it via diffusion will have a higher chance of being multiplied and detected by the circuitry, which explains why the enhancement of PDP is expected in our new proposed device designed with the double multiplication region technique.

### Measurement techniques

In the light emission tests, the device was biased without any quenching circuit to see the light emission, and the images were taken in the dark by means of a digital microscope (Hirox KH-8700) connected to a charge-coupled device (CCD) camera. To operate the device in Geiger mode, it was quenched and recharged passively via an externally connected 660 kilo-ohms resistor to the SPAD’s anode while the bias voltage was applied to the cathode node. The generated pulses on the resistor were then counted with a high-speed digital oscilloscope (Teledyne LeCroy WavePro 760Zi-A) at 50% of the peak magnitude. To estimate the percentage of afterpulsing in this study, we made use of the inter-arrival time method, where the time intervals are measured between subsequent pulses generated by the SPAD^33^. To measure the PDP of the device in a wide spectrum, a broad-band Xenon light source was utilized. Thanks to a monochromator, each target wavelength was then selected via diffraction gratings. The incident power was attenuated with an aperture whose size can be controlled with a micrometer. Spatially uniform illumination was achieved via an integrating sphere. Also, to measure the precise incident light power, we used a calibrated silicon photodiode (Hamamatsu S2281). The timing jitter of the device was acquired by time-correlated single-photon counting (TCSPC), where a pulsed monochromatic laser was used, whose 100 kHz clock signal served as a reference. A digital oscilloscope recorded the timing histogram, while the single-photon regime was ensured through neutral density filters.

## Data Availability

The datasets used and/or analysed during the current study available from the corresponding author on reasonable request.
